# Equipment, measurement and dose—a survey for therapeutic ultrasound

**DOI:** 10.1186/s40349-016-0051-1

**Published:** 2016-03-02

**Authors:** Adam Shaw, Eleanor Martin, Julian Haller, Gail ter Haar

**Affiliations:** Acoustics and Ionizing Radiation Division, National Physical Laboratory, Teddington, Middlesex UK; Present address: Biomedical Ultrasound Group, University College London, London, UK; Physikalisch-Technische Bundesanstalt, Braunschweig, Germany; Division of Radiotherapy and Imaging, Institute for Cancer Research, Sutton, Surrey UK

**Keywords:** Therapeutic ultrasound, Dosimetry, Exposimetry, Survey

## Abstract

**Background:**

Dosimetry for Ultrasound Therapy (DUTy) is a large international project which addresses the development of a metrological infrastructure for the determination of ultrasound exposure and dose to tissue.

**Methods:**

In order to seek the views of the wider therapy ultrasound community and to review dose and in situ exposure quantities that have been suggested or used previously, a web-based questionnaire containing a range of questions covering the type of ultrasound equipment that is used and the range of applications for which it has been developed was created at www.surveymonkey.com. This questionnaire was intended to cover any contemporary therapeutic ultrasound application (including physiotherapy, lithotripsy and drug delivery) and asked specific questions about quantification of in situ exposure and dose, especially as relevant to treatment planning, standardisation and/or regulation.

**Results:**

This paper summarises the 123 responses submitted between February and September 2014 to the questions on clinical applications, equipment, quality assurance (QA) and measurement and standards, as well as to those relating to an understanding of “dose” in the context of ultrasound. The full set of anonymous responses is available in an additional Excel file.

**Conclusions:**

The results clearly demonstrate the need not only for further improvements in measuring devices and for measurement guidelines but also for a wider dissemination and higher awareness of existing standards. Whilst it is unlikely that a single definition of dose can be sufficient for all ultrasound treatment modalities, the answers clearly indicate that many aspects would benefit from clear definitions of relevant dose quantities and shed light on the preferred form of such definitions.

**Electronic supplementary material:**

The online version of this article (doi:10.1186/s40349-016-0051-1) contains supplementary material, which is available to authorized users.

## Background

This work forms part of a project entitled Dosimetry for Ultrasound Therapy (DUTy) which is supported in part by the European Metrology Research Programme, jointly funded by the EMRP participating countries within EURAMET and the European Union. The project is coordinated by the National Physical Laboratory (UK) in partnership with eight other institutes (www.duty-project.eu). The aim of the project is to address the metrological infrastructure for the determination of ultrasound exposure and dose to tissue. One of its tasks required a review of the in situ exposure and dose quantities that have been suggested, or previously used, and sought to investigate the views of the wider therapy ultrasound community.

This paper summarises the 123 responses submitted to questions about clinical applications, equipment, QA and measurement and standards, as well as to questions related to understanding of “dose” in the context of ultrasound. The full set of anonymous responses is available in an additional Excel file [see Additional file 1]. Many of the questions relating to applications, equipment and measurement are similar to those in an earlier survey carried out by two of the current authors [1, 2]. A companion paper [3] covers in detail the questions on ultrasound dose from the present survey and also discusses a proposed framework for exposure and dose quantities: readers with an interest in the dose aspects are advised to refer to this earlier paper.

## Methods

The set of questions was intended to help give a picture of the R&D currently going into therapeutic ultrasound. It covered equipment and measurements related to any contemporary therapeutic ultrasound applications (including, for instance, physiotherapy, lithotripsy and drug delivery).

The survey was advertised through the International Society of Therapeutic Ultrasound (ISTU) mailing list, by the Focused Ultrasound Foundation, by notices at the ISTU 2014 conference in Las Vegas and by emails to personal contacts of the DUTy team members. It was divided into four main sections which are as follows:

- *Your therapeutic ultrasound application* (Q1–Q11)—this asked about the applications of therapeutic ultrasound with which the respondents had significant involvement and about the transducers and acoustic fields with which they worked.
- *Measurements you currently perform* (Q12–Q24)—this was primarily related to the transducer input/output characteristics and the acoustic field generated in water.
- *Your measurement needs* (Q25–Q26)—this asked about the respondents’ perception of the shortcomings of the acoustic field measurements they currently make and what their priorities for future improvements were.
- *Your opinion about dose* (Q27–39)—this enquired about the application of the concept of “dose” to therapeutic ultrasound.

However, the breakdown of results presented here is grouped slightly differently, in terms of respondents, applications, equipment used, QA and measurement, standards and dose. Detailed questions about specific exposure and dose quantities are not covered here, but they are available in the full dataset in the additional Excel file [see Additional file 1] and are presented in a separate paper [3] which also discusses a proposed framework for exposure and dose quantities.

## Results

The results for most of the questions are presented in this section. The complete questions as well as the possible answers and their response percentages can be found in the additional Excel file [see Additional file 1]. The additional file also contains a few questions that are not mentioned in this section.

### Respondents

There were 123 responses, with 90 completing the questionnaire fully, including the final questions on dose. The respondents covered most disciplines with approximately 30 % in each of biological effects research and systems development, and a further 10 % in measurement or QA; 5 % were clinical users, 5 % involved with standards/regulation and 5 % in modelling/treatment planning. The remaining 15 % were involved in “other” areas (Q42). There was a wide range in length of experience, with responses being quite evenly spread between 2 and 20 years (Q41); nearly half of the replies were from the USA (34 %) and UK (13 %), with the remaining respondents being from a total of 13 other countries including France, Germany and China (Q40).

### Applications

**Fig. 1:**
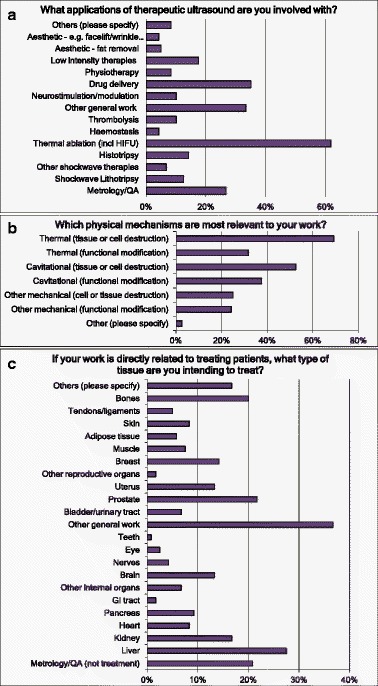
Responses to questions Q1 (**a**), Q4 (**b**) and Q2 (**c**) related to clinical applications. Multiple answers were possible (“Please tick all that apply”)

**Fig. 2:**
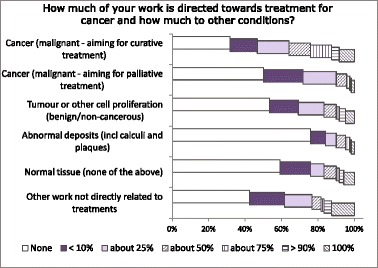
Responses to question Q3. Each respondent’s total does not have to add up to 100 %

The most common application area (Q1) was thermal ablation (including high-intensity focused ultrasound (HIFU)) followed by drug delivery; next were generic areas not related to a specific treatment (for example, general field modelling or biological interaction mechanisms) and metrology/QA (Fig. 1a). The major mechanisms (Q2) thought to be involved were cell destruction by heat and by cavitation (Fig. 1b), but non-lethal cell modification was also substantial. Within those that did work on specific treatments, many tissue types were represented (Fig. 1c). We asked how much time was directed towards curative treatment of cancer and other categories of treatment (Q3): on average, 29 % of the effort went towards curative cancer treatment with 12 % giving palliative treatment and a further 15 % working with benign tumours (Fig. 2).

### Equipment used

**Fig. 3:**
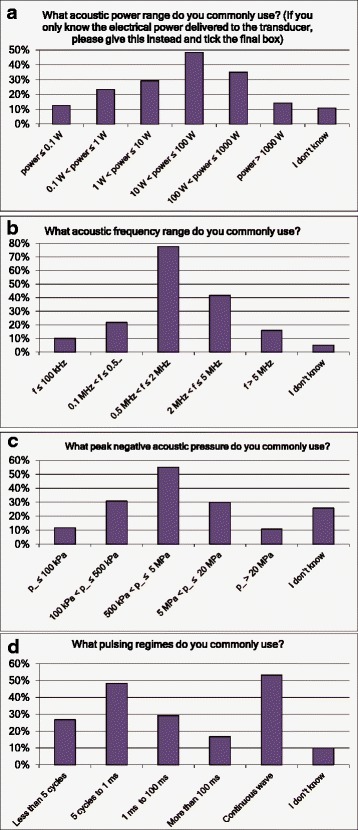
Responses to questions Q5 to Q8 (**a**–**d**) related to equipment characteristics. Multiple answers were possible (“Please tick all that apply”)

**Fig. 4:**
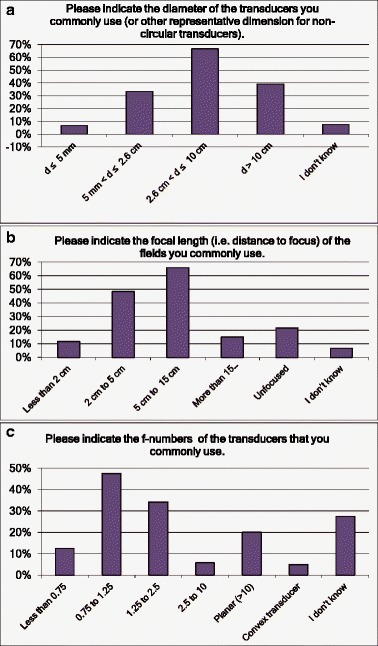
Responses to questions Q9 to Q11 (**a**–**c**) related to equipment characteristics. Multiple answers were possible (“Please tick all that apply”)

Questions were asked about the range of ultrasound characteristics for equipment used: output power, frequency, negative pressure, pulsing, transducer diameter, focal length and F-number. The responses are summarised in Figs. 3 and 4: the typical system had a transducer with a diameter between 2 and 10 cm (Q9), focused at 5 to 10 cm (Q10) and operating at frequencies between 0.5 and 2 MHz (Q6). Output power was between 10 and 100 W (Q5) with a peak negative pressure between 0.5 and 5 MPa (Q7) and with either a continuous wave or a burst length between 5 cycles and 1 ms (Q8). These results clearly demonstrate the great diversity of the ultrasound sources employed for therapeutic purposes and, therefore, the complexity of appropriate characterisation for this range of sources.

### QA and measurement

**Fig. 5:**
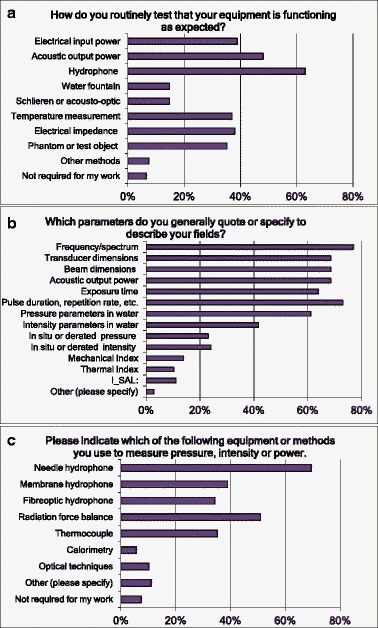
Responses to questions Q13 to Q15 (**a**–**c**) related to measurements made. Multiple answers were possible (“Please tick all that apply”)

The equipment used for measurement under different circumstances, the parameters measured, the phantoms and biological models used and the approach taken were also of interest: these questions and responses are shown in Fig. 5. For acoustic measurements such as pressure, intensity or power, 46 % of respondents said they made measurements at, or close to, typical settings for use and 28 % made measurements at lower settings and extrapolated to high levels (Q16). For measurements of heating, cavitation or other effects, an even higher percentage (69 %) measured at, or close to, typical settings for use (Q18).

**Fig. 6:**
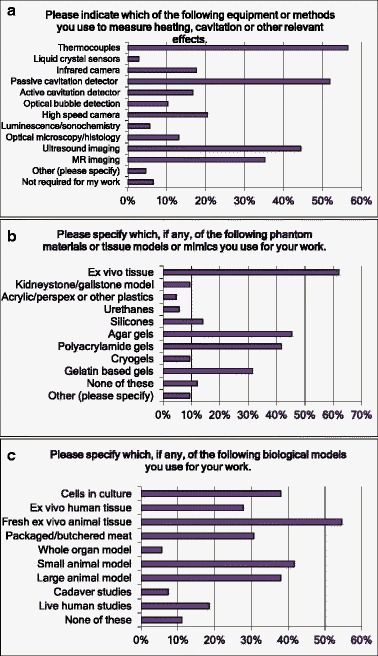
Responses to questions Q17 (a), Q19 (b) and Q20 (c) related to the measurements made. Multiple answers were possible (“Please tick all that apply”)

As might be expected, fresh ex vivo animal tissue is the most common biological model used (Fig. 6) followed equally by small and large animal models and cell cultures (Q20). One in six respondents was involved with human studies. Ex vivo tissue also tops the non-biological models ahead of agar, polyacrylamide and gelatine gels (Q19).

**Fig. 7:**
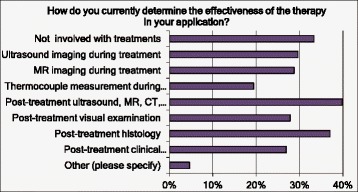
Responses to questions Q21 related to the determination of the therapeutic effectiveness. Multiple answers were possible (“Please tick all that apply”)

We also asked about how the effectiveness of a therapy was assessed (Q21): post-treatment imaging (MR, CT or ultrasound) was the most widely used, closely followed by histology (Fig. 7).

### Standards

**Fig. 8:**
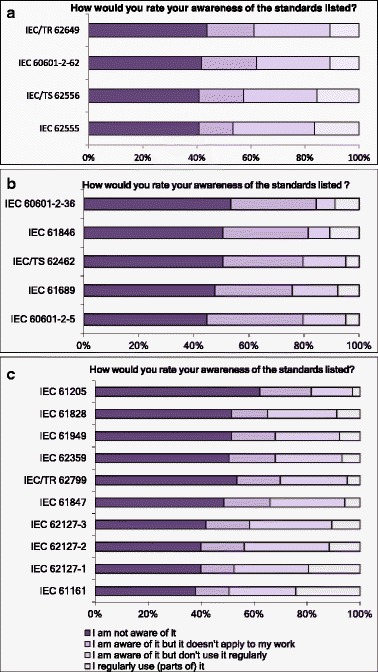
Responses to questions Q22 to Q24 (**a**–**c**) about the awareness of IEC standards related to ultrasound

Recently, two new IEC Standards (IEC 60601-2-62 [4], IEC 62555 [5]) and a technical specification (IEC 62556 [6]) have been published, following a strategy laid out in an earlier technical report (IEC 62649 [2]). In order to test awareness of these documents, the respondents were asked about these (Fig. 8a, Q22) and other IEC publications relevant to some aspects of medical ultrasound (Fig. 8b and c, Q23–Q24). The full list of the standards listed, including their titles, is given in Table 1. The highest numbers for awareness and regular use were found for IEC 61161 and 62127-1. This correlates with the answers to two other questions we asked about routine tests (Q13) and measurement equipment (Q15), where hydrophone measurements (63 %) and output power measurements (48 %) were found to be the most common measurements, and the most common measurement devices were needle hydrophones (69 %) and radiation force balances (51 %).

**Table 1:** Full list of the standards given in the questionnaire

Number	Type	Name	Ed.	Year	Topic
60601-2-62 [4]	S	Medical electrical equipment - Part 2-62: Particular requirements for the basic safety and essential performance of high intensity therapeutic ultrasound (HITU) equipment	1.0	2013	HITU
62555 [5]	S	Ultrasonics - Power measurement - Output power measurement for high intensity therapeutic ultrasound (HITU) transducers and systems	1.0	2013	HITU
62556 [6]	TS	Ultrasonics - Field characterization - Specification and measurement of field parameters for high intensity therapeutic ultrasound (HITU) transducers and systems	1.0	2014	HITU
62649 [2]	TR	Requirements for measurement standards for high intensity therapeutic ultrasound (HITU) devices	1.0	2010	HITU
60601-2-5 [7]	S	Medical electrical equipment - Part 2-5: Particular requirements for the basic safety and essential performance of ultrasonic physiotherapy equipment	3.0	2009	PT
61689 [8]	S	Ultrasonics - Physiotherapy systems - Field specifications and methods of measurement in the frequency range 0,5 MHz to 5 MHz	3.0	2013	PT
62462 [9]	TS	Ultrasonics - Output Test - Guide for the maintenance of ultrasound physiotherapy systems	1.0	2007	PT
61846 [10]	S	Ultrasonics - Pressure pulse lithotripters - Characteristics of fields	1.0	1998	LT
60601-2-36 [11]	S	Medical electrical equipment - Part 2-36. Particular requirements for the safety of equipment for extracorporeally induced lithotripsy	2.0	2014	LT
61161 [12]	S	Ultrasonics - Power measurement - Radiation force balances and performance requirements	3.0	2013	Gen
62127-1 [13]	S	Ultrasonics - Hydrophones - Part 1: Measurement and characterization of medical ultrasonic fields up to 40 MHz	1.1	2013	Gen
62127-2 [14]	S	Ultrasonics - Hydrophones - Part 2: Calibration for ultrasonic fields up to 40 MHz	1.1	2013	Gen
62127-3 [15]	S	Ultrasonics - Hydrophones - Part 3: Properties of hydrophones for ultrasonic fields up to 40 MHz	1.1	2013	Gen
61847 [16]	S	Ultrasonics - Surgical systems - Measurement and declaration of the basic output characteristics	1.0	1998	Gen
62799 [17]	TR	Models for evaluation of thermal hazard in medical diagnostic ultrasonic fields	1.0	2013	Gen
62359 [18]	S	Ultrasonics - Field characterization - Test methods for the determination of thermal and mechanical indices related to medical diagnostic ultrasound fields	2.0	2010	Gen
61949 [19]	S	Ultrasonics - Field characterization - In situ exposure estimation in finite-amplitude ultrasonic beams	1.0	2007	Gen
61828 [20]	S	Ultrasonics - Focusing transducers - Definitions and measurement methods for the transmitted fields	1.0	2001	Gen
61205 [21]	S	Ultrasonics - Dental descaler systems - Measurement and declaration of the output characteristics	1.0	1993	Other

*S* full Standard, *TS* technical specification, *TR* technical report, *HITU* high-intensity therapeutic ultrasound, *PT* physiotherapy, *LT* lithotripsy, *Gen* general medical ultrasound

**Fig. 9:**
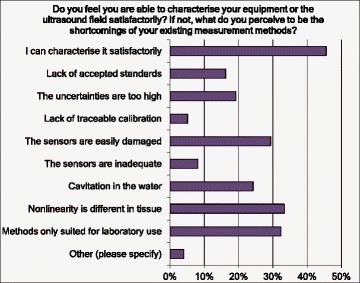
Responses to question Q25 about perceived shortcomings of current measurement methods

Questions were also asked about the perceived shortcomings of the measurement methods being used (Fig. 9) as this should indicate where improvements are likely to be of most benefit in future standards and guidelines (Q25). It is noteworthy that less than 50 % of the respondents felt that they could characterise their equipment satisfactorily. The answers clearly indicate that the development of more robust sensors and/or sensors for use in clinical systems, and of guidelines for measurements in situ (i.e. not in water), or for nonlinear derating, should be of highest priority.

Another question asked (Q12) was “Is there a particular exposure geometry in your application that makes acoustic field measurement difficult?” Forty-six percent said that the geometry for their work was straightforward, but the other answers indicated that future work should be directed towards measurements in acoustic fields with multiple foci or complex focal distributions (22 %), multiple independent beams (15 %) and specially formed transducers (14 %) (such as intraluminal or intravascular devices): the need to measure close to the transducer surface or “inside the bowl” was also noted.

### What is “dose”?

**Fig. 10:**
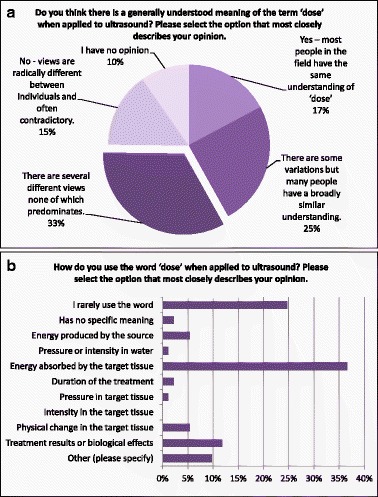
Responses to questions Q27 (**a**) and Q28 (**b**) about general and personal usage of the word “dose”

Within this section of the questionnaire, the participants were asked about their own usage of the term “dose” with respect to therapeutic ultrasound (Q28) as well as whether they thought that it has a generally understood meaning (Q27). Although responses about the general understanding were widely spread with “There are several different views none of which predominates” being the most common answer (33 %, Fig. 10a), the question about the respondents’ own understanding of the term yielded a clear “winner”, as more than one third (36 %) chose “It means something quantifiable about the amount of energy absorbed by the target tissue” as the answer that most closely described their opinion (Fig. 10b).

In agreement with that, the two potential definitions based on energy (“Absorbed energy per unit mass” (Q36) and “Applied total acoustic energy” (Q37)) were always among the top three, when rated against relevance to the respondents’ own application, relevance to other applications and acceptability (Q34–Q39, see Additional file 1 or [3] for further details). When “Familiarity” was assessed, “Absorbed energy per unit mass” yielded the highest percentage on “Seen in other literature”, indicating that this term is much more common in radiotherapy and the quantification of electromagnetic waves. This preference for “energy” was also reflected by the answers to Q33, where “Acoustic power” and “Exposure time” (whose product is energy) were both believed to be important for the effectiveness of their application by 79 % of the respondents.

For all of the four criteria mentioned above, “Thermally equivalent time” was the most popular option—52 % claimed to “Use it regularly” under the “Familiarity” section, 40 % found it “Very relevant” when asked about its “Relevance to my own application”, 52 % thought that it was “Generally acceptable” and 30 % assumed that it was “Relevant to most” when asked to comment on “Relevance to other applications”. For the last category, “Applied total acoustic energy” had a slightly higher score (34 %).

The strong relationship between the understanding of the term “dose” and thermal mechanisms is also shown by the answers to Q29 (86 % thought dose (as they understood it) is relevant to effects mediated by thermal mechanisms) and Q32, where 70 % thought that “thermal mechanisms” should be of highest priority for the development of future standards.

**Fig. 11:**
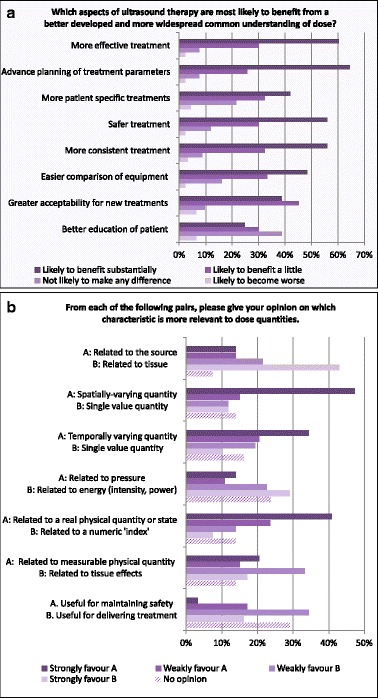
Responses to questions Q30 (**a**) and Q31 (**b**) about possible benefits of a better understanding of “dose” and possible characteristics of “dose”

The participants were given a further list of eight aspects of ultrasound therapy with the request to score whether these would benefit “substantially” or “a little”, or are “likely to become worse” from a common understanding of “dose” (Fig. 11a). Most aspects received the highest responses for “Likely to benefit substantially” (with 64 % for “Better planning of treatment parameters in advance” being the highest). The exceptions were “Greater acceptability for new treatments” (where 45 % selected “Likely to benefit a little”) and “Better education of patient before treatment” (where 38 % selected “Not likely to make any difference”).

As a step on the route towards a common definition of “dose”, the participants were given pairs of characteristics for which they were asked to favour one (Fig. 11b).

## Discussion 

The majority of the questions in the first three sections were straightforward to understand and to answer. The answers on the topic of acoustic conditions used on the one hand not only show the challenging great diversity of conditions (from *P* < 0.1 W to *P* > 1000 W, from *f* < 0.1 MHz to *f* > 5 MHz, from *p*_r_ < 0.1 MPa to *p*_r_ > 20 MPa, from less than five wave cycles to continuous wave) but also clearly indicated the most common regimes for most of the quantities, on which further development of standards, definitions and measuring devices should focus.

However, the section about dose was more problematic because, although the word “dose” is widely used in different areas of medicine, it is not always used in exactly the same way. This makes it difficult to decide what is most appropriate for ultrasound therapy and to compare responses on an equal basis. It was necessary, therefore, to provide some guidance to respondents whilst trying not to bias the answers in any way. The respondents were given descriptions of the usage of “dose” based on wording from Wikipedia entries:

- In biochemistry, dose is the quantity of something (chemical, physical, or biological) that may impact an organism biologically; the greater the quantity, the larger the dose.
- In medicine, the term is usually applied to the quantity of a drug or other agent administered for therapeutic purposes.
- In toxicology, dose may refer to the amount of a harmful agent (such as a poison, carcinogen, mutagen, or teratogen), to which an organism is exposed.
- For ionising radiation absorbed dose is a measure of the energy deposited in a medium by ionizing radiation per unit mass of the medium.

It is not necessary for there to be a single-dose quantity which is suitable for all therapeutic ultrasound applications. For instance, dose for lithotripsy may be very different to dose for physiotherapy. The participants were asked specifically about several quantities which are sometimes used in ultrasound but which are dimensionally very different:

- Total acoustic energy output (SI unit: joule)
- Absorbed energy per unit mass (SI unit: joule/kg)
- Intensity-time-integral (SI unit: joule/m^2^)
- Local cavitation index (SI unit: dimensionless or MPa/√MHz)
- Thermally equivalent time (commonly called “thermal dose” or “cumulative equivalent minutes at 43 °C”) (SI unit: s)

The responses to these questions are covered in detail in a separate paper [3], but some basic results will be briefly discussed here as well. The two quantities which are most used by the participants were thermally equivalent time and total applied energy, and these were also the quantities that were seen as being of most direct relevance to the individual’s own work and also of most general relevance. They were also, therefore, the quantities likely to be most acceptable to the wider community. Absorbed energy per unit mass was generally the next highest scoring, but it should be noted that this quantity (coupled to the thermal properties of the tissue, local blood flow and other aspects) is what governs the temperature rise and therefore the thermally equivalent time. So, although not directly important in itself, knowledge of these distributions is a critical step in planning a treatment.

### Summary and conclusions

This paper summarises the 123 responses submitted to online survey questions about therapeutic ultrasound, which was prepared as part of the EMRP project “Dosimetry for Ultrasound Therapy” and hosted at www.surveymonkeycom. There were questions on clinical applications, equipment, QA and measurement and standards, as well as some questions related to understanding of the term “dose” in the context of ultrasound. The full set of anonymised responses is available as an additional Excel file and a separate paper [3] has been published which discusses exposure and dose in more detail and presents a framework for underpinning future standards and improved metrology in this area.

A clear result from this survey is that less than 50 % of the respondents felt that they could characterise their equipment satisfactorily, clearly demonstrating the need for further improvements in measuring devices and for measurement guidelines.

Another finding is that, among the respondents, only 50 % are aware of the relevant standards dealing with therapeutic ultrasound devices or ultrasound in general. On the other hand, an encouraging 45 % of the respondents expressed their willingness to participate in future development of standards (Q43).

Another result worth emphasising is that the answers clearly indicate that the respondents assume that many aspects would benefit from clear definitions of dose quantities for therapeutic ultrasound (Q30). Concerning the particular “form” or characteristics of such a definition, it is a noteworthy result that for all pairs in Q31, a clear “winner” was obtained. On balance, there was a preference for dose to be a spatially and temporally variable quantity which is related to absorption of energy in the exposed medium. Its role should be more directed towards improving treatment than in addressing safety concerns. Only the pair “Related to measurable physical quantity” vs. “Related to tissue effects” was unclear, perhaps reflecting the lack of traceable quantities that are biologically based.

### Availability of supporting data

The data set supporting the results of this article is included within the article’s additional file:

- File name: “Shaw et al. 2015—Additional File 1.xlsx”
- File format: Microsoft Office Excel,.xlsx
- Title of data: Full set of anonymous responses to the survey
- Description of data: The file contains an overview sheet and 44 sheets for the 44 questions of the survey

## Additional file

Additional file 1: Full set of anonymous responses to the survey. The file contains an overview sheet and 44 sheets for the 44 questions of the survey. (XLSX 154 kb)

## References

[1] Shaw A, Ter Haar G (2006). Requirements for measurement standards in high intensity focused ultrasound (HIFU) fields.

[2] International Electrotechnical Commission: “IEC/TR 62649 Requirements for measurement standards for high intensity therapeutic ultrasound (HITU) devices”, Edition 1.0, Geneva, Switzerland, 2010

[3] Shaw A, Ter Haar G, Haller J, Wilkens V (2015). Towards a dosimetric framework for therapeutic ultrasound. Int J Hyperth.

[4] International Electrotechnical Commission: “IEC 60601-2-62 Medical electrical equipment - Part 2-62: Particular requirements for the basic safety and essential performance of high intensity therapeutic ultrasound (HITU) equipment”, Edition 1.0, Geneva, Switzerland, 2013

[5] International Electrotechnical Commission: “IEC 62555 Ultrasonics - Power measurement - Output power measurement for high intensity therapeutic ultrasound (HITU) transducers and systems”, Edition 1.0, Geneva, Switzerland, 2013

[6] International Electrotechnical Commission: “IEC/TS 62556 Ultrasonics - Field characterization - Specification and measurement of field parameters for high intensity therapeutic ultrasound (HITU) transducers and systems”, Edition 1.0, Geneva, Switzerland, 2014

[7] International Electrotechnical Commission: “IEC 60601-2-5 Medical electrical equipment - Part 2-5: Particular requirements for the basic safety and essential performance of ultrasonic physiotherapy equipment”, Edition 3.0, Geneva, Switzerland, 2009

[8] International Electrotechnical Commission: “IEC 61689 Ultrasonics - Physiotherapy systems - Field specifications and methods of measurement in the frequency range 0,5 MHz to 5 MHz”, Edition 3.0, Geneva, Switzerland, 2013

[9] . International Electrotechnical Commission: “IEC/TS 62462 Ultrasonics - Pressure pulse lithotripters - Characteristics of fields”, Edition 1.0, Geneva, Switzerland, 1998

[10] International Electrotechnical Commission: “IEC 61846 Medical electrical equipment - Part 2-62: Particular requirements for the basic safety and essential performance of high intensity therapeutic ultrasound (HITU) equipment”, Edition 1.0, Geneva, Switzerland, 2013

[11] International Electrotechnical Commission: “IEC 60601-2-36 Medical electrical equipment - Part 2-36. Particular requirements for the safety of equipment for extracorporeally induced lithotripsy”, Edition 2.0, Geneva, Switzerland, 2014

[12] International Electrotechnical Commission: “IEC 61161 Ultrasonics - Power measurement - Radiation force balances and performance requirements”, Edition 3.0, Geneva, Switzerland, 2013

[13] International Electrotechnical Commission: “IEC 62127-1 Ultrasonics - Hydrophones - Part 1: Measurement and characterization of medical ultrasonic fields up to 40 MHz”, Edition 1.1, Geneva, Switzerland, 2013

[14] International Electrotechnical Commission: “IEC 62127-2 Ultrasonics - Hydrophones - Part 2: Calibration for ultrasonic fields up to 40 MHz”, Edition 1.1, Geneva, Switzerland, 2013

[15] International Electrotechnical Commission: “IEC 62127-3 Ultrasonics - Hydrophones - Part 3: Properties of hydrophones for ultrasonic fields up to 40 MHz”, Edition 1.1, Geneva, Switzerland, 2013

[16] International Electrotechnical Commission: “IEC 61847 Ultrasonics - Surgical systems - Measurement and declaration of the basic output characteristics”, Edition 1.0, Geneva, Switzerland, 1998

[17] International Electrotechnical Commission: “IEC/TR 62799 Models for evaluation of thermal hazard in medical diagnostic ultrasonic fields”, Edition 1.0, Geneva, Switzerland, 2013

[18] International Electrotechnical Commission: “IEC 62359 Ultrasonics - Field characterization - Test methods for the determination of thermal and mechanical indices related to medical diagnostic ultrasound fields”, Edition 2.0, Geneva, Switzerland, 2010

[19] International Electrotechnical Commission: “IEC 61949 Ultrasonics - Field characterization - In situ exposure estimation in finite-amplitude ultrasonic beams”, Edition 1.0, Geneva, Switzerland, 2007

[20] International Electrotechnical Commission: “IEC 61828 Ultrasonics - Focusing transducers - Definitions and measurement methods for the transmitted fields”, Edition 1.0, Geneva, Switzerland, 2001

[21] International Electrotechnical Commission: “IEC 61205 Ultrasonics - Dental descaler systems - Measurement and declaration of the output characteristics”, Edition 1.0, Geneva, Switzerland, 1993

